# Vortex-electromagnetic-wave-based ISAR imaging for high-speed maneuvering targets

**DOI:** 10.1038/s41598-022-22185-1

**Published:** 2022-10-26

**Authors:** Lijun Bu, Yongzhong Zhu, Yijun Chen, Yufei Yang, Yadan Zang

**Affiliations:** College of Information Engineering, Engineering University of PAP, Xi’an, 710086 China

**Keywords:** Electrical and electronic engineering, Information theory and computation

## Abstract

Vortex electromagnetic wave (VEMW) carrying orbital angular momentum (OAM), which is expected to introduce additional degrees of freedom in inverse synthetic aperture radar(ISAR) imaging. However, the current research about maneuvering targets is based on the "stop go" hypothesis, which does not apply to high-speed motion scenarios due to the intrapulse movement of the target. To improve the imaging quality, this letter proposes a VEMW-based high-speed maneuvering targets imaging method. Firstly, the ISAR imaging scenario of high-speed target is established. According to the spatial geometric relationship between radar and maneuvering target, the vortex echo is deduced and its characteristics are analyzed. Subsequently, a frequency modulation rate estimation method considering both calculation efficiency and high precision is proposed to realize the accurate estimation of target speed. Then, an adaptive azimuth image compensation method based on minimum entropy is proposed. Through the setting of threshold, the number of component signals in linear frequency modulation (LFM) signal is determined and compensated successively. Finally, the range profile and azimuth profile are combined to reconstruct the three-dimensional information. The simulation results demonstrate that this work can effectively eliminate the influence of high-speed motion on range and azimuth profile, also benefit the development of ISAR imaging technique of high-speed maneuvering targets.

## Introduction

With the development of science and technology in recent years, ISAR imaging for non-cooperative targets, has been extensively studied by scholars at home and abroad because of its wide application in military and civil fields. The mechanism of ISAR imaging is to use the Doppler information generated by the target’s rotation to distinguish the echoes returned from different parts of the target. Therefore, when the relative motion between the target and the radar is insufficient, in order to improve the cross-range resolution of the traditional planar electromagnetic wave ISAR, it is necessary to transmit more pulses or a longer coherent processing interval (CPI). In this way, a large number of echo data will be generated in the imaging process, which will not only cause great pressure on the radar system, but also affect the real-time imaging of the target. For high-speed maneuvering targets, it will also bring problems such as phase tracking error and Doppler ambiguity.

In the past 30 years, due to unique spiral phase structure and orthogonal characteristics^1^, vortex electromagnetic wave has attracted increasing attention in the fields of rotation Doppler parameter estimation^2,3^, OAM modulated wireless communication^4–6^, quantum application^7^. In addition, when the moving target is irradiated by vortex radar, the unique wavefront shows the characteristic of angular diversity, the dual relationship between the OAM mode number and the azimuth angle, so the scattered echo in the target area will contain more relevant information, which make the electromagnetic vortex radar is expected to develop into a new imaging mechanism.

In 1992, Allen et al.^8^ first discovered the Laguerre Gauss beam had a spatial spiral wavefront, and also determines the relationship between the wavefront structures and intrinsic values of OAM. In 2013, Guo et al. established a scattering point imaging model based on VEMW via the uniform circular array (UCA), and explained the relationship between OAM mode value and azimuth by using the spectrum characteristics of Bessel function, which proved the potential of vortex radar imaging^9^. Since then, domestic researchers have put forward a variety of motion imaging models of vortex radar and corresponding algorithms for these models, which realize the vortex imaging of moving targets. By analysing the relationship between the rotation Doppler and azimuth,then compensating the echo signal range profile, the azimuth imaging of rotating disk is realized^10^. A new method of vortex imaging based on improved back projection (BP) algorithm is proposed^11^. The influence of additional terms of vortex electromagnetic wave on azimuth phase is compensated by increasing azimuth modulation function. Besides, it also revealed the relationship between azimuth resolution and OAM mode number. The traditional synthetic aperture radar (SAR) imaging and the vortex radar are combined^12^, through adding Bessel term compensation and azimuth filtering in the traditional RD algorithm, the two-dimensional imaging of moving targets is realized, which demonstrates that the combined vortex imaging method has more advantages in azimuth resolution. The vortex radar is first applied to ISAR imaging^13^, the traditional Fast Fourier Transform (FFT) algorithm is replaced by the convolution back projection (CBP) and Power Spectrum Density (PSD) estimation, and the combination of CBP and PSD estimation is better than FFT from the simulation results. To improve the anti noise performance, an electromagnetic vortex imaging method based on the generation of fractional OAM beams is proposed^14^, which show that the method has strong noise resistance and effectively overcomes the shortcomings of poor imaging performance of low-order OAM beam.

From the current research results, most of the moving imaging of vortex radar is based on the "stop go" model, and the target motion speed is relatively slow. But with the development of high-speed aircraft, the speed of air target continues to increase. The existing supersonic aircraft can fly at more than five times the speed of sound^15^. Therefore, the intrapulse motion of high-speed target can not be ignored, and the problems such as range migration, Doppler dimension expansion and azimuth blur are also unavoidable^16^. To solve these problems, Ref.^17^ establish the imaging model of high-speed of ISAR and propose a joint estimation method for echo signal. Through the reconstruction of the target azimuth, the 3-D target imaging is finally realized. But the joint estimation method has its limitations. On the one hand, it has high precision to the model constructed in this paper; on the other hand, the estimated velocity still has some error with the actual velocity, which makes the constructed compensation phase inaccurate. Above all, the reference only considers the case that the Y-axis coordinate difference of scattering point is small, that is, when the value of error phase term is close, the common compensation method is used to realize the image quality improvement. But the target with great difference in position distribution is not studied in depth.

In this context, a VEMW-based high-speed maneuvering targets ISAR imaging method is proposed. Firstly, based on the papers published earlier^17^, this paper makes an in-depth analysis on the targets with large differences in the position distribution of scattering points. After that, through the frequency modulation rate estimation, the phase function is constructed to compensate the echo signal. Then the compensated phase error term is estimated by the least squares quadratic polynomial regression method (LSPR), so as to further accurately estimate the frequency modulation rate of the echo signal and the speed of moving target. Finally, according to the characteristics of a multi-component LFM signal, this paper carries out two-dimensional search and estimation of azimuth parameters based on the minimum entropy method and realizes adaptive compensation of component signals through the setting of threshold. Finally, the deterioration of range-azimuth imaging is overcome and simulation results realize three-dimensional information reconstruction.

The rest of this paper is organized as follows. In section “Vortex ISAR imaging model”, the imaging scenario is introduced and echo signal model is derived. In section “Imaging processing algorithm”, the compensation algorithm of high-speed movement is described in detail. The simulation experiment is carried out and the imaging results are analyzed in section “Simulation and analysis”. The conclusions are summarized in section “Conclusion”.

## Vortex ISAR imaging model

### ISAR imaging model

**Figure 1:**
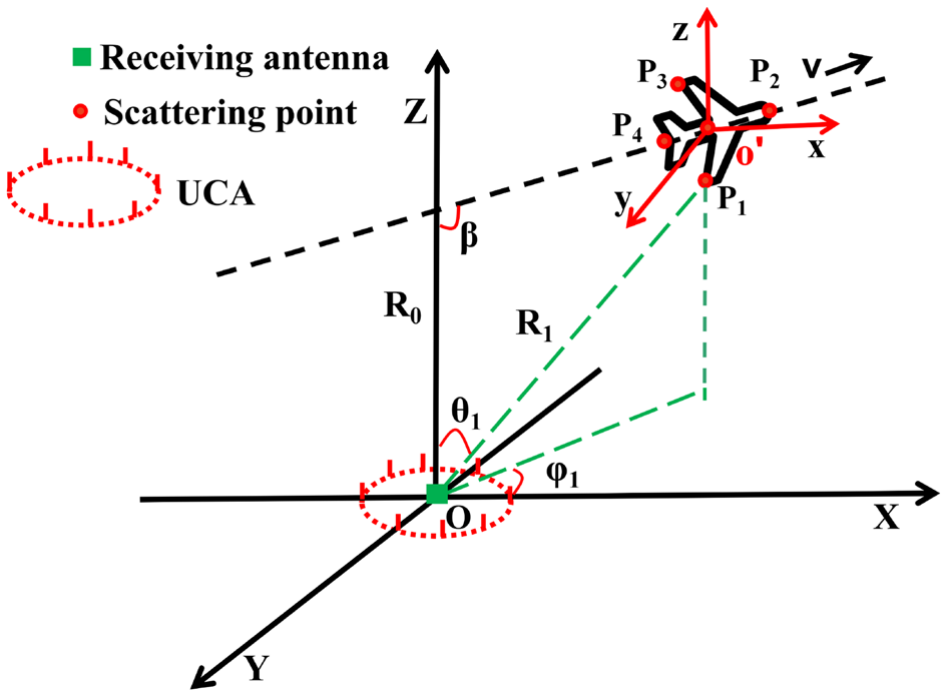
Geometry of the 3-D vortex ISAR imaging.

In this section, the imaging scenario for vortex radar ISAR is described and the signal model is deduced. As shown in Fig. 1, the vortex radar system can be realized by UCA^18,19^, and the center is located at *O*(0, 0, 0) in the global coordinate system. The target with multiple scattering points moves at the speed *v* towards the direction of the angle $\beta$ with the *Z*-axis, the path of motion is in the XOZ plane and the intercept of this direction on the positive *Z*-axis is $R_{0}$. The origin $o'$ of the reference coordinate system is also the equivalent phase center. The global radar coordinate system $O-XYZ$ and reference coordinate $o'-xyz$ are parallel to each other. For a scattering point $P_b(x_b,y_b,z_b)$ $b\in 1,2,\ldots ,T$ on the target in the coordinate $o'-xyz$, its position in $O-XYZ$ coordinate system is

1 $$\begin{aligned} \begin{aligned} x_B&=v_xt_m+x_b\\ y_B&=y_b\\ z_B&=R_{0}+\frac{v_{x} t_{m}}{\tan (\beta )}+z_{b} \end{aligned} \end{aligned}$$

Then the instantaneous range between the radar and the scattering point $P_b$ is $R_{Pb}=\sqrt{x_{B}^{2}+y_{B}^{2}+z_{B}^{2}}$. The angle between the line connecting the scattering point and radar center O and the positive *Z*-axis is the pitch angle $\theta$. The azimuth $\varphi$ refers to the angle between the projection direction of the scattering point on the *XOY* plane and the positive *X*-axis.

### Vortex signal model

Assuming that the LFM signal is utilized to generate vortex EM waves^20^, and the emission signal of each UCA element can be expressed as

2 $$\begin{aligned} \begin{aligned} s\left( t, t_{m}, l\right) ={\text {rect}}\left( \frac{t}{T_{P}}\right) \cdot \exp \left( i 2 \pi f_{c} t+i \pi K t^{2}\right) \cdot \exp \left( i l \phi _{n}\right) \end{aligned} \end{aligned}$$

where $t_m$,*t*,$T_P$ and $f_c$ are the slow time, fast time, pulse width and carrier frequency, respectively. *K* is the linear frequency modulation ratio and ${\text {rect}}({\mathrm {t}} / {\mathrm {Tp}})$ is the range envelop. *l* is the OAM mode value, and the modulation phase of the $n-th$ element is $\phi _{n}=n \frac{2 \pi }{N}, {\mathrm {n}}=0,1,2, \ldots , {\mathrm {N}}-1$.

3 $$\begin{aligned} \begin{aligned} s\left( t, t_{m}, l\right)&=\sum _{b=1}^{T} \sigma _{b}(r, \theta , \varphi ) {\text {rect}}\left( \frac{t-\tau _{b}}{T_{P}}\right) \cdot \exp \left[ i 2 \pi f_{c}\left( t-\tau _{b}\right) \mathrm{{ }}+i \pi K\left( t-\tau _{b}\right) ^{2}\right] \cdot \exp \left( i l \varphi _{Pb}\right) \end{aligned} \end{aligned}$$

where $\sigma _{b}(r, \theta , \varphi )$ donates backscattering coefficient, and $\tau _{b}=2 R_{Pb} / c$ represents the instantaneous range delay. To reduce sampling frequency, the dechirping processing is adopted here, and the reference signal is constructed as follows:

4 $$\begin{aligned} \begin{aligned} s_{r e f}(t)={\text {rect}}\left( \frac{t-\tau _{r e f}}{T_{P}}\right) \exp \left[ i 2 \pi f_{c}\left( t-\tau _{r e f}\right) +i \pi K\left( t-\tau _{r e f}\right) ^{2}\right] \end{aligned} \end{aligned}$$

where $r_{r e f}$ represents the distance from the equivalent phase center o’ to the radar center O, $\tau _{r e f}=2 r_{r e f} / c$ is the corresponding delay. The dechirping process can be regarded as the conjugate multiplication of (3) and (4). After simplification, the echo signal is:

5 $$\begin{aligned} \begin{aligned} s_{m i x}\left( t_{s}, t_{m}, l\right)&= s\left( t, t_{m}, l\right) \cdot s_{r e f}^{*}(t) \\&= \sum _{b=1}^{T} \sigma _{b} r e c t\left( \frac{t_{s}-\tau _{\Delta b}}{T_{P}}\right) \exp \left[ -i 2 \pi \left( f_{c} \tau _{\Delta b}-\frac{1}{2} K \tau _{\Delta b}^{2}+K \tau _{\Delta b} t_{s}\right) \right] \exp \left[ i l \varphi _{Pb}\left( t_{s}\right) \right] \end{aligned} \end{aligned}$$

where $\tau _{\Delta b}=\tau _{b}-\tau _{r e f}$ is the relative echo delay of the $b-th$ scattering point, $t_{s}=t-\tau _{r e f}$ is to change the zero time of fast time. When the target moves at high speed, the radial movement in single pulse time reaches the wavelength level, so the intrapulse Doppler effect should be considered. Assuming the velocity of the target is *v*, the instantaneous distance of the scattering point $P_b$ is $R_{Pb}\left( t_{s}\right) =R_{Pb}\left( t_{m}\right) +v t_{s}$, then the relative echo delay is $\tau _{\Delta b}\left( t_{s}\right) =\tau _{\Delta b}+\frac{2 v}{c} t_{s}$. The azimuth of scattering point $P_b$ in coordinate axis $O-XYZ$ is $\varphi _{Pb}=\arctan \frac{y_{B}}{x_{B}}$, and the second order Taylor expansion is

6 $$\begin{aligned} \begin{aligned} \varphi _{Pb}\left( t_{s}\right)&=\arctan \frac{y_{b}}{x_{b}+v_{x}t_{s}} \\&\approx \varphi _{Pb}(0)+\frac{\partial \varphi _{Pb}\left( t_{s}\right) }{\partial t_{s}} t_{s} \\&\approx \arctan \frac{y_{b}}{x_{b}}-\frac{v_{x} y_{b}}{x_{b}^{2}+y_{b}^{2}} t_{s} \end{aligned} \end{aligned}$$

The first-order coefficients c1 of the fitted curve expression can be obtained by the above analysis and processing.

7 $$\begin{aligned} \begin{aligned} c_{1}=-\frac{v_{x} y_{b}}{x_{b}^{2}+y_{b}^{2}} \end{aligned} \end{aligned}$$

**Figure 2:**
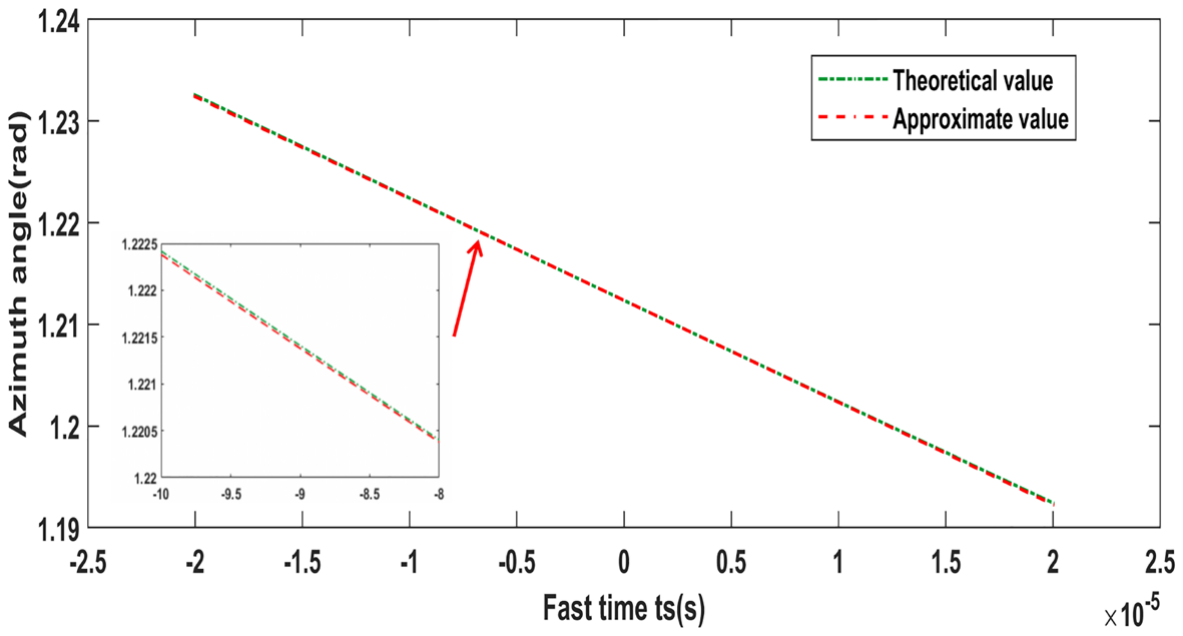
The comparison between the theoretical values of the instantaneous azimuth and Taylor series expansion.

Figure 2 exhibits the comparison between the Taylor approximation and the theoretical value of the instantaneous azimuth expression. The results show that the theoretical value is basically consistent with the Taylor approximation, indicating that a good fitting effect has been achieved.

According to the above analysis, the echo signal after dechirping process is written as

8 $$\begin{aligned} \begin{aligned} s_{m i x}\left( t_{s}, t_{m}, l\right)&\approx \sum _{b=1}^{T} \sigma _{b} r e c t\left( \frac{t_{s}-\tau _{\Delta b}\left( t_{s}\right) }{T_{P}}\right) \\&\quad \cdot \exp \left[ -i 2 \pi \left( f_{c} \tau _{\Delta b}^{2}-\frac{1}{2} K \tau _{\Delta b}^{2}+K \tau _{\Delta b} t_{s} \right) \right] \\&\quad \cdot \exp \left[ -i 2 \pi \left( 2 f_{c} v / c-2 K v \tau _{\Delta b} / c-\frac{v_{x} y_{b}}{x_{b}^{2}+y_{b}^{2}} \right) t_{s}\right] \\&\quad \cdot \exp [-i 2 \pi (2 K v / c \left. \left. -2 K v^{2} / c^{2}\right) t_{s}^{2}\right] \\&\quad \cdot \exp \left[ i 2 l\left( \varphi _{Pb, t_{m}}(0)\right) \right] \end{aligned} \end{aligned}$$

As shown in (8), because of the high-speed movement of the multi scattering points in the pulse duration, the signal processed by dechirping is the superposition of multi-component LFM signals with the same frequency and different center frequency. For the convenience of subsequent analysis, it can be rewritten as follows:

9 $$\begin{aligned} \begin{aligned} s_{m i x}\left( t_{s}, t_{m}, l\right)&\approx \sum _{b=1}^{T} \sigma _{b} {\text {rect}}\left( \frac{t_{s}-\tau _{\Delta b}\left( t_{s}\right) }{T_{P}}\right) \exp \left( i \psi _{b}+i f_{b} t_{s}+i K^{*} t_{s}^{2}\right) \exp \left[ i 2 l \varphi _{Pb, t_{m}}(0)\right] \end{aligned} \end{aligned}$$

The first term $\psi _{b}$ is consistent with the phase expression under the "stop go" model in (5). The second term is the initial frequency of the multi-component LFM signal, and the third one is frequency modulation ratio. Because they are functions of time $t_s$, the broadening of range profile and the azimuth ambiguity will be caused during imaging. In order to realize the accurate estimation of multi-component LFM signal parameters to compensate the deterioration of imaging quality, the following algorithm is proposed.

## Imaging processing algorithm

**Figure 3:**
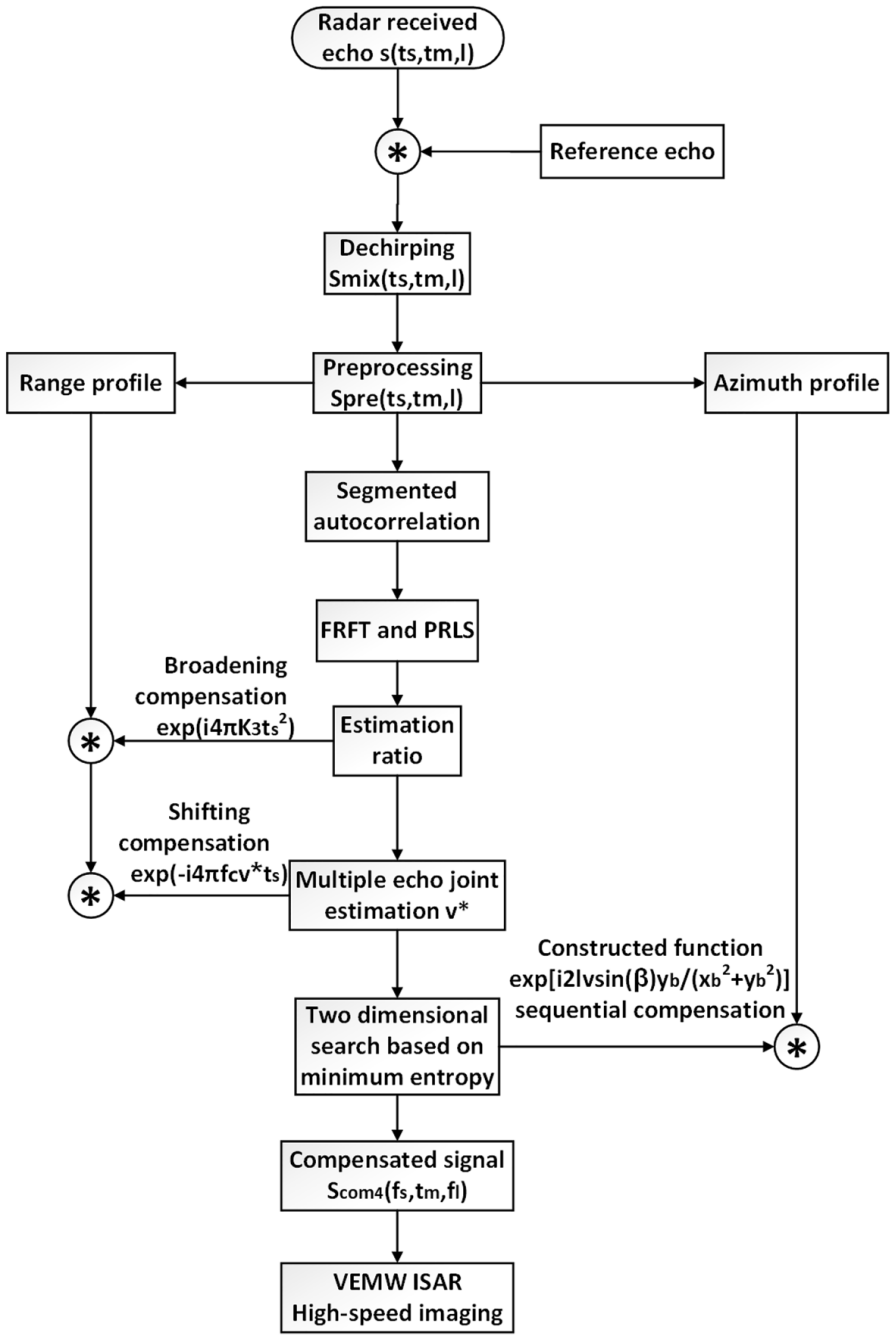
The flowchart of the proposed vortex imaging algorithm.

The flow chart of the imaging algorithm is shown in Fig. 3. Firstly, the baseband echo signal after dechirping operation is preprocessed to form a simple signal expression in pulse time. Then, the ratio ${\mathrm {K}}_2$ is roughly estimated by using the method of piecewise autocorrelation and fractional Fourier transformation (FRFT), and the quadratic phase function is constructed to compensate. The error term $\delta K$ is estimated by LSPR, and the precise estimation of the ratio ${\mathrm {K}}_3$ is obtained by combining ${\mathrm {K}}_2$ and $\delta K$. Then moving speed of the target can be obtained by ${\mathrm {K}}_3$. To enhance the estimation accuracy, the velocity *v* of multiple echos estimation can effectively eliminate variation error. After that, the parameters of azimuth profile are obtained by two-dimensional search, and the corresponding entropy is calculated. According to the principle of minimum entropy, the original signal is reconstructed and the estimated component signal is removed, and the ambiguity in azimuth profile is solved. Finally, the deterioration of two dimensional imaging is improved, and the 3D image is obtained according to the spatial geometric relationship.

According to the estimation method proposed in Ref.^17^, the echo signals at different times are autocorrelated to find the maximum frequency under different sequence differences, and the rough frequency modulation value ${\mathrm {K}}_1$ is obtained.At this time, although the calculation efficiency of ${\mathrm {K}}_1$ value is very high, the accuracy is low, and further estimation is needed. Then, taking ${\mathrm {K}}_1$ as the reference value, FRFT is used to estimate the ratio ${\mathrm {K}}_2$ with a small step value within the value range obtained. The expression of the ratio ${\mathrm {K}}_2$ is as follows:

10 $$\begin{aligned} \begin{aligned} K_{2}=\frac{\tan \left[ \left( \alpha _{i}-1\right) \pi / 2\right] f_{s}^{2}}{N_{i}} \end{aligned} \end{aligned}$$

where $N_{i}$ is the number of sampling points for the fast time $t_{s}$, and $f_{s}$ is the corresponding time-domain discrete sampling frequency. At this time, although the ratio ${\mathrm {K}}_2$ value has been obtained, there is still a certain error with the real value $K^{*}$. In order to further improve the accuracy and efficiency of the estimation, ${\mathrm {K}}_2$ obtained from (10) can be used as a reference to preliminarily compensate the echo signal. Then LSPR is used for exquisite estimation, so as to effectively improve the estimation effect.

The phase compensation function $\exp \left( -i \pi {\mathrm {K}}_2 {\mathrm {ts}}^{2}\right)$ is constructed, and the compensated signal expression is:

11 $$\begin{aligned} \begin{aligned} s_{c o m 1}\left( t_{s}, t_{m}, l\right)&= s_{m i x}\left( t_{s}, t_{m}, l\right) \cdot \exp \left( -i \pi K_{2} t_{s}^{2}\right) \\&\approx \sum _{b=1}^{T} \sigma _{b} r e c t\left( \frac{t_{s}-\tau _{\Delta b}\left( t_{s}\right) }{T_{P}}\right) \exp \left[ i 2 l\varphi _{Pb, t_{m}}(0)]\right] \exp \left( i \psi _{b}+i f_{b} t_{s}+i \pi \delta K t_{s}^{2}\right) \end{aligned} \end{aligned}$$

where $\delta K=K^{*}-K_{2}$ represents the error term between the true value $K^{*}$ and ${\mathrm {K}}_2$, and obtain the phase information ${\text {ph}}={\text {imag}}\left( s_{\text{ com } 1}\right) / {\text {real}}\left( s_{\text{ com } 1}\right)$. According to the echo data at different slow times, error curve is fitted by LSPR and the error term $\delta K$ is calculated. Then the precise estimation value of the ratio ${\mathrm {K}}_{3}=K_{2}+\delta K$, and use ${\mathrm {K}}_3$ to recompensate the quadratic term in (8). The compensated echo is as follows:

12 $$\begin{aligned} \begin{aligned} s_{\text{ com } 2}\left( t_{s}, t_{m}, l\right)&= s_{m i x}\left( t_{s}, t_{m}, l\right) \cdot \exp \left( -i \pi K_{3} t_{s}^{2}\right) \\&\approx \sum _{b=1}^{T} \sigma _{b} r e c t\left( \frac{t_{s}-\tau _{\Delta b}\left( t_{s}\right) }{T_{P}}\right) \cdot \exp \left( i \psi _{b}+i f_{b} t_{s}\right) \exp \left( i 2 l \varphi _{Pb, t_{m}}(0)\right) \end{aligned} \end{aligned}$$

As shown in (12), the broadening of range profile has been eliminated by the compensation of quadratic term. Subsequently, in order to eliminate the influence of the coefficient of the first-order term, the relevant parameters need to be estimated according to the above ratio ${\mathrm {K}}_3$. Then according to the relationship between (8) and (9), the estimated frequency modulation rate $K^{*}$ satisfies

13 $$\begin{aligned} \begin{aligned} K^{*}=-2 \pi \left( 2 K v / c-2 K v^{2} / c^{2}\right) \end{aligned} \end{aligned}$$

In order to effectively eliminate variation error, multiple echoes are combined to estimate the movement velocity v. Assuming that the total number of echoes transmitted by radar is M, the estimated velocity can be represented by one-dimensional vector v

14 $$\begin{aligned} v=\left[ \begin{array}{llll} v_{1},&\quad v_{2}, \ldots ,&\quad v_{k}, \ldots ,&\quad v_{M} \end{array}\right] \end{aligned}$$

where $v_{k}$ represents the estimated speed of the k-th echo. On this basis, the obtained average speed value is:

15 $$\begin{aligned} \begin{aligned} v^{*}=\frac{\sum _{i=1}^{M_{p u l}}\left[ c \cdot \left( \frac{K_{i}^{*}+\pi K}{4 \pi K}\right) ^{1 / 2}+\frac{c}{2}\right] }{M_{p u l}} \end{aligned} \end{aligned}$$

According to (8), the corresponding compensation phase term is constructed to compensate (12), and the signal form can be expressed as

16 $$\begin{aligned} \begin{aligned} s_{\text{ com3 } }\left( t_{s}, t_{m}, l\right)&=s_{\text{ comp } 2}\left( t_{s}, t_{m}, l\right) \cdot \exp \left[ i\left( 4 \pi f_{c} v^{*} / c\right) t_{s}\right] \\&\approx \sum _{b=1}^{T} \sigma _{b} r e c t\left( \frac{t_{s}-\tau _{\Delta b}\left( t_{s}\right) }{T_{P}}\right) \exp \left( i 2 l \varphi _{Pb, t_{m}}(0)\right) \exp \left( i \psi _{b}+i f_{w}^{*} t_{s}\right) \end{aligned} \end{aligned}$$

where $f_{w}^{*}=4 \pi K v \tau _{\Delta P} / c-2 l \frac{v \sin \beta }{y_{b} / \tan ^{2}\left( \varphi _{Pb}(0)\right) +y_{b}}$.The broadening of range profile has been eliminated, and only the error phase term of azimuth needs to be compensated, and fuzzy interval of azimuth can be obtained by imaging. The value range of $y_b$ can be obtained through priori information. When the constructed compensation term is close to the real value, the entropy is the minimum. Then the entropy vector E is depicted as follows:

17 $$\begin{aligned} \begin{aligned} E=\left[ E_{d q 1}, E_{d q 2}, \ldots , E_{d q j}, \ldots , E_{d q N}\right] \end{aligned} \end{aligned}$$

where $E_{d q j}$ represents the j-th entropy vector when the azimuth is d-th search and the $y_b$ is q-th search. However, the azimuth Angle of each scatter point on the target is different, so it can’t complete the estimation of all the scatter points in one search, and each scatter point needs to be compensated iteratively. In the initial stage, after the azimuth image compensation of the first scatter, the result with the lowest entropy value is used as the benchmark parameter for the first iteration compensation. Then the reference parameters are extended by the principle of minimum entropy variance to search for the azimuth image with the best compensation effect. Then the second iteration is carried out according to the results of the second search, and the benchmark parameters are updated. In this way, the number of benchmark parameters is gradually increased until the variance of entropy value is less than the threshold value. The variance $D_E$ of entropy vector is calculated, and its mathematical expression is as follows:

18 $$\begin{aligned} \begin{aligned} D_{E}=\sum _{j=1}^{N}\left( E_{d q j}-\bar{E}\right) ^{2} / N^{*} \end{aligned} \end{aligned}$$

where $\bar{E}=\sum _{j=1}^{N} E_{d q j} / N$ represents the mean value of entropy vector $\bar{E}$. $\varepsilon$ is the threshold to determine whether the phase of the primary term exists or not. If $D_{E} \ge \varepsilon$, there is still component signal in the remaining signal, and continue to search compensation. Otherwise, the compensation of signal is completed and the parameter estimation will be finished. The signal after the last compensation is:

19 $$\begin{aligned} \begin{aligned} s_{\text{ com } 4}\left( t_{s}, t_{m}, l\right)&=s_{\text{ comp3 } }\left( t_{s}, t_{m}, l\right) \cdot \exp \left( -i f_{b} t_{s}\right) \\&\approx \sum _{b=1}^{T} \sigma _{b} {\text {rect}}\left( \frac{t_{s}-\tau _{\Delta ^{b}}\left( t_{s}\right) }{T_{P}}\right) \exp \left[ i \psi _{b}+i\left( 4 \pi K v \tau _{\Delta b} / c\right) t_{s}\right] \exp \left[ i 2 l \varphi _{Pb, t_{m}}(0)\right] \end{aligned} \end{aligned}$$

Performing FFT on (19) in fast time domain $t_s$ and the OAM domain, then we obtain echo signal after compensating residual video phase (RVP) and oblique term.

20 $$\begin{aligned} \begin{aligned} s_{c o m 4}\left( f_{s}, t_{m}, f_{l}\right)&=\sum _{b=1}^{T} \sigma _{b} \sin c\left[ T_{P}\left( f_{s}+K \tau _{\Delta b}\left( 1-\frac{2 v}{c}\right) \right] \right. \cdot \exp \left( -i 2 \pi 
f_{c} \tau _{\Delta b}\right) \cdot P\left[ f_{l}-\varphi _{Pb, t_{m}}(0) / \pi \right] \end{aligned} \end{aligned}$$

Since the speed of light *c* is much greater than the maneuvering speed *v*, so $1-\frac{2 v}{c} \approx 1$, and where $P\left( f_{l}\right)$ is the envelope of azimuth profile. At this time, the compensation for the deterioration of imaging quality caused by high-speed motion has been completed, and the $y_b$ of each scattering point are obtained by the minimum entropy method. And the moving speed v of the target can be obtained by Eq. (13). Then combined with the coefficient value c1 of the first-order term obtained after fitting, we can get the abscissa of the scattering point. The Z-axis coordinates are obtained from the distance image information before. Finally, the objective of 3D information reconstruction.

**Table 1:** Key parameters in the simulation.

Parameters	Values	Unit
Central frequency $f_c$	9	GHz
Pulse width $T_P$	80	$\upmu \mathrm{{s}}$
Bandwidth *B*	4	GHz
Pulse repetition frequency	1000	Hz
Signal to noise ratio (SNR)	− 5	dB
Scene height $R_0$	8	Km
Flight speed *v*	2339	m/s
Inclination $\beta$	150	$^{\circ }$
OAM mode $l_{max}$	50	–
Relative coordinates of $$P_1 $$	(0, $$-\sqrt{1.8 } / 2$$, 1)	m
Relative coordinates of $$P_2 $$	(0, 0, 1.15)	m
Relative coordinates of $$P_3 $$	(0, $$\sqrt{1.8 } / 2$$, 1.3)	m
Relative coordinates of $$P_4 $$	(0, 0.5, 0.85)	m
Relative coordinates of $$P_5 $$	(0, − 0.5, 0.7)	m

**Figure 4:**
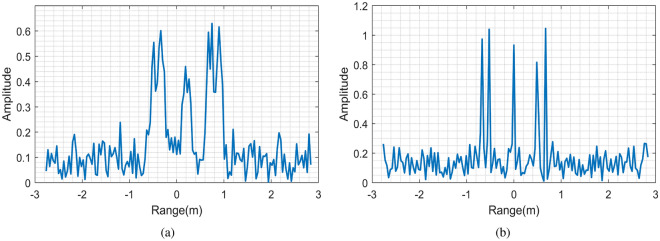
Comparison of range profiles. (**a**) Range profile before compensation. (**b**) Compensation for range profile broadening.

## Simulation and analysis

This section is used to verify the effectiveness of the proposed algorithm and its superiority in LFM signal parameter estimation compared with other references. The simulation parameters are set as shown in Table 1.

Figure 4a shows the range profile distribution obtained by FFT in the fast time domain. It is obvious that the range profile is broadened and shifted seriously caused by the high-speed movement.

In order to eliminate this phenomenon, 500 echo signals are extracted to estimate the ratio ${\mathrm {K}}_1$ by using the method of segment autocorrelation. The estimation results are shown in Table 2. It can be seen that the method has obvious advantages in calculation efficiency, but the accuracy is low. The search step is 0.0001 and the setting range is $\alpha _K \pm 0.01$, then 500 echo signals are still extracted for experiment and the ratio is ${\mathrm {K}}_2=-3.1646 \times 10^{7}$. The corresponding compensation function $\exp \left( -i \pi K_{2} t_{s}^{2}\right)$ is constructed, and the phase information after compensation is obtained.

As shown in Table 2, the results of fitting error terms by using LSPR for 500 echo phases, wherein the quadratic coefficient $\delta {\mathrm {K}}=1.9792 \times 10^{7}$, the precise estimate of the ratio ${\mathrm {K}}_{3}=\delta {\mathrm {K}}+{\mathrm {K}}_{2}=-3.1448\times 10^{9}$, the specific results are shown in Table 2.

**Table 2:** Performance comparison of estimation methods.

Method	True ratio	Estimated ratio	Error (%)	Execution time (s)	Velocity (m/s)	Velocity error (%)
Autocorrelation ${\mathrm {K}} 1$	$-\,3.1187\times 10^{9}$	$-\,2.9119\times 10^{9}$	6.63	1.5320	2183.9	6.63
Reference^17^ ${\mathrm {K}} 2$	$-\,3.1187\times 10^{9}$	$-\,3.1646\times 10^{9}$	1.47	6.8923	2451.8	4.82
Proposed method ${\mathrm {K}} 3$	$-\,3.1187\times 10^{9}$	$-\,3.1448\times 10^{9}$	0.84	7.0514	2373.3	1.47

**Figure 5:**
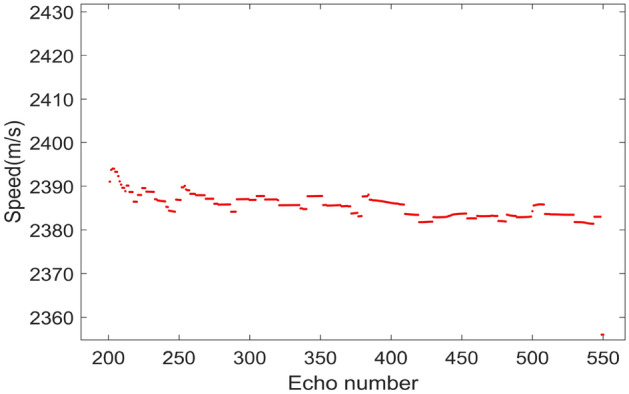
Velocity distribution under 350 echoes.

After obtaining the precise estimation of frequency modulation ratio, Fig. 5 shows the velocity distribution under 350 echoes according to (13), and the average velocity ${\mathrm {v}}^{*}=2373.3\,{\mathrm {m}} /{\mathrm {s}}$, with an error of 1.47$\%$. Then one-dimensional range profile is compensated and the results are shown in Fig. 4b. Compared with Fig. 4a, the broadening and shift of range profile have been improved well.

**Figure 6:**
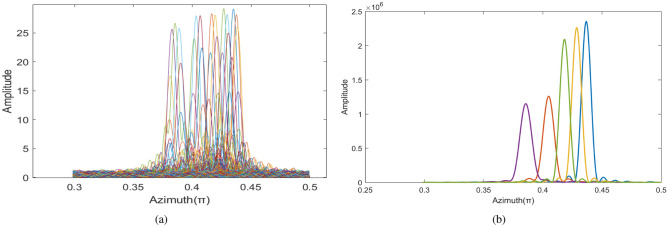
Comparison of azimuth profiles. (**a**) Uncompensated azimuth profile. (**b**) Azimuth profile after compensation.

Then, the ambiguity of azimuth profile needs to be compensated. As shown in Fig. 6a, the image of the fifth echo is performed after FFT transformation in OAM domain. The range of azimuth ambiguity is $[0.3706 \pi , 0.4531 \pi ]$, and the corresponding Shannon entropy can be obtained from envelope calculation is 7.4616.

By searching for different azimuth angles and $y_b$ according to the above information, the corresponding compensation function is constructed to compensate the echo in turn, and the variance $D_{E}$ of entropy vector *E* can be calculated. According to the results of many experiments, the threshold of this paper $\varepsilon$ set to $2\times 10^{-5}$.

After 5 compensations of echo signal, the entropy value is 7.0228 and the variance is $1.74\times 10^{-5}$, which is less than threshold and can terminate the cycle. Figure 6b shows the azimuth profile of the target after the high-speed motion compensation is completed. Compared with Fig. 6a, the ambiguity of the azimuth profile is greatly improved, which also proves the effectiveness of the proposed method.

**Figure 7:**
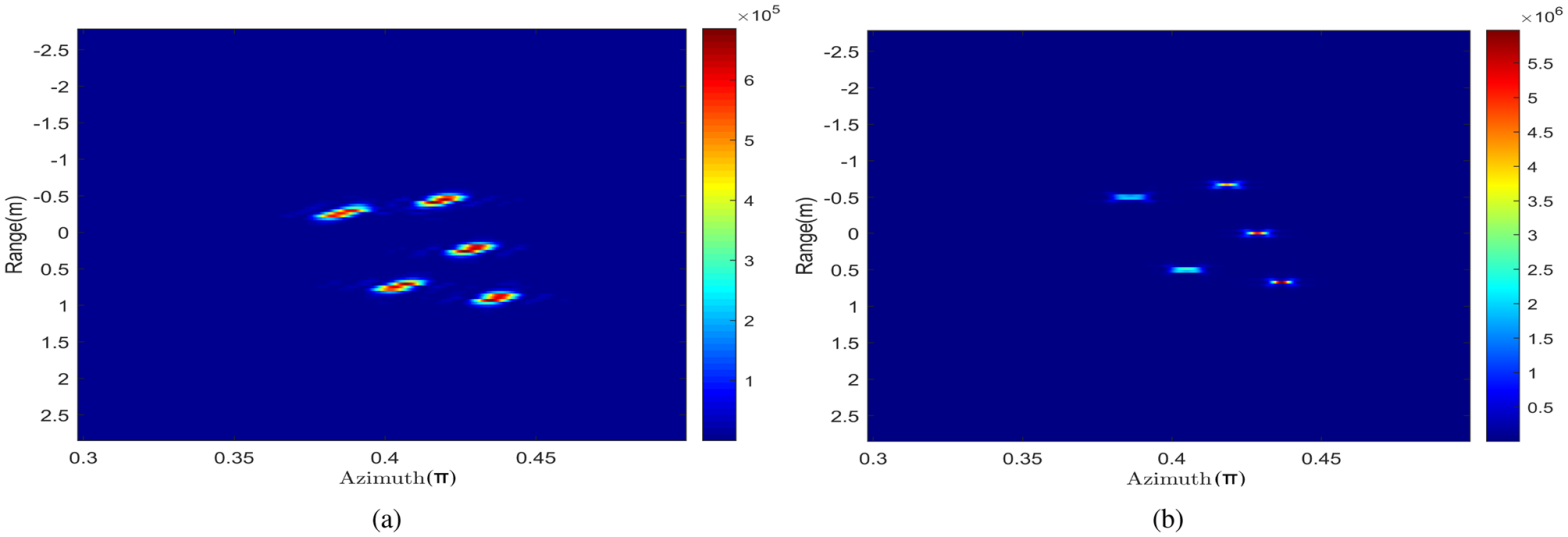
Comparison of two-dimensional images. (**a**) Two-dimensional images before compensation. (**b**) Compensated two-dimensional images.

**Table 3:** Coordinates of each scattering point of the target.

Scattering point information	Theoretical coordinates	Average error (%)
Scattering point $P_1$	(0.014, − 0.672, 0.995)	0.609
Scattering point $P_2$	(− 0.015, 0, 1.151)	1.391
Scattering point $P_3$	(0.02, 0.672, 1.298)	0.973
Scattering point $P_4$	(0.031, 0.485, 0.85)	1.185
Scattering point $P_5$	(− 0.013, − 0.523, 0.699)	4.417

**Figure 8:**
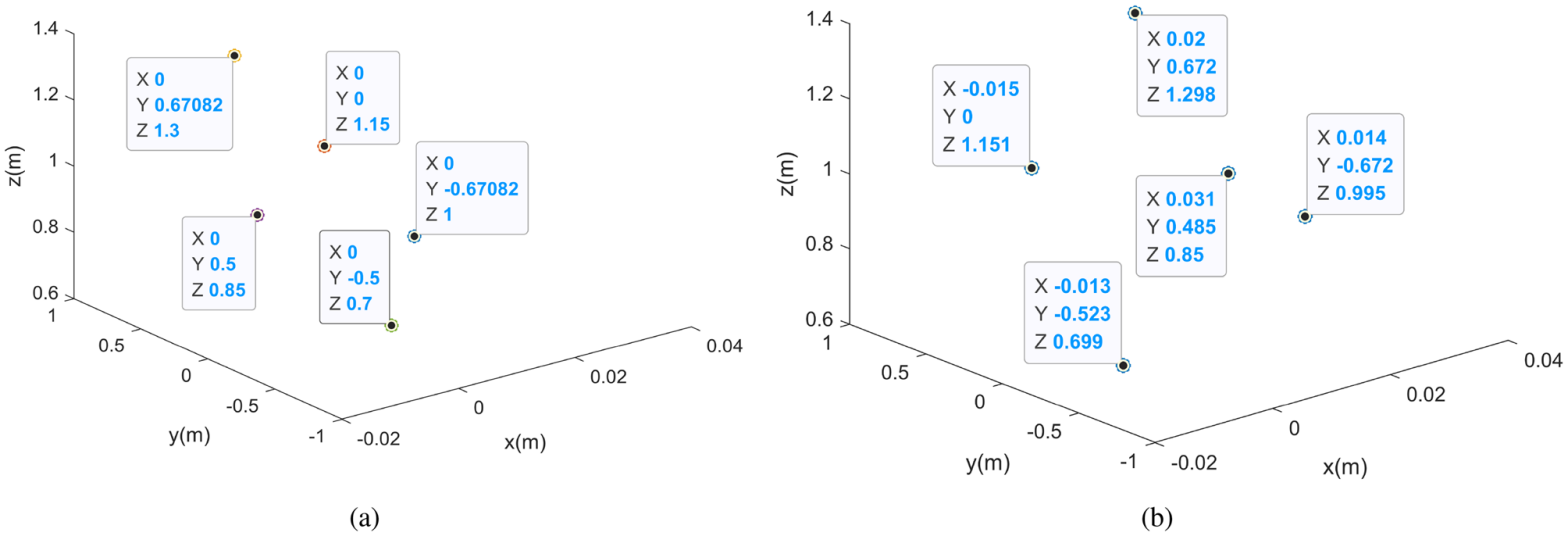
Comparison of 3D imaging results of point targets. (**a**) Ground truth. (**b**) Reconstructed imaging result.

Figure 7b is the two-dimensional imaging result of the compensated range-azimuth. Compared with Fig. 7a, it can be seen that the imaging quality have been improved better, and the anti-noise performance of the proposed imaging algorithm is good.

Finally, the reconstruction information of each scattering point can be obtained, as shown in Table 3. Compared with the real value of the target, the error is small. Figure 8 shows the final 3D information reconstruction of the target.

**Figure 9:**
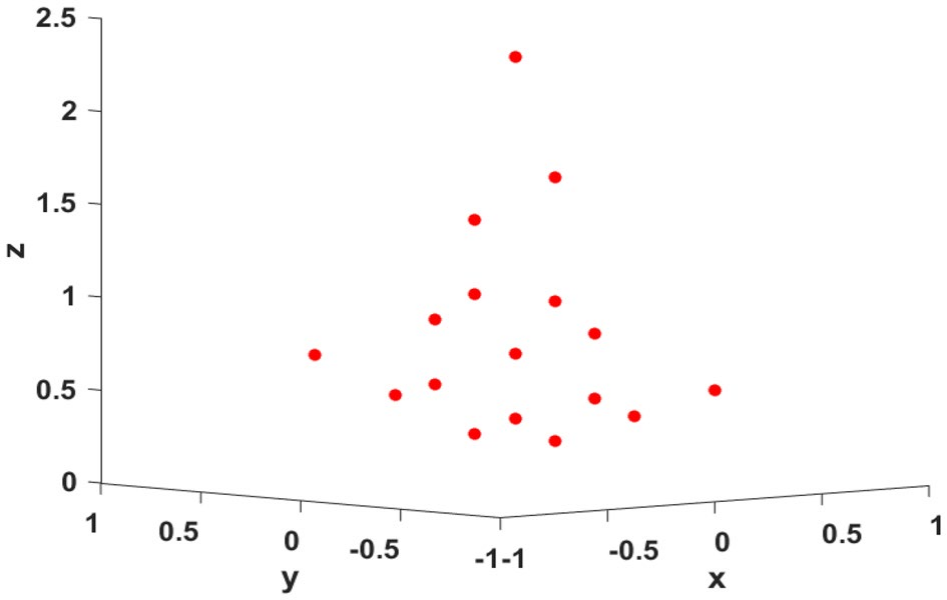
Ground truth.

**Figure 10:**
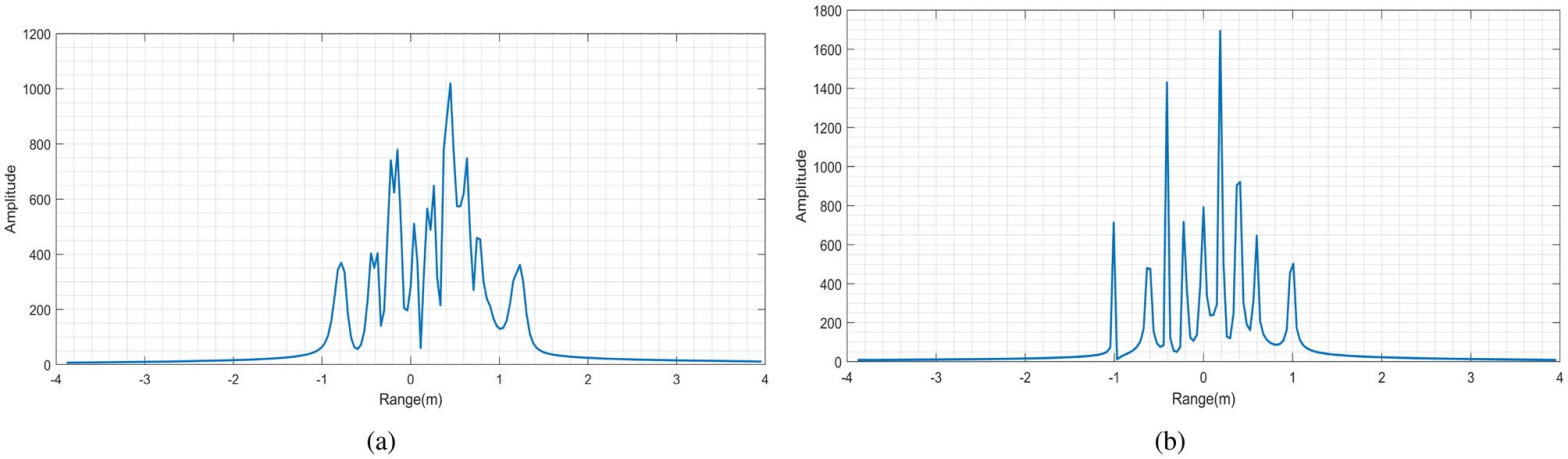
Range profile. (**a**) Range profile before compensation. (**b**) Compensated range profile.

**Figure 11:**
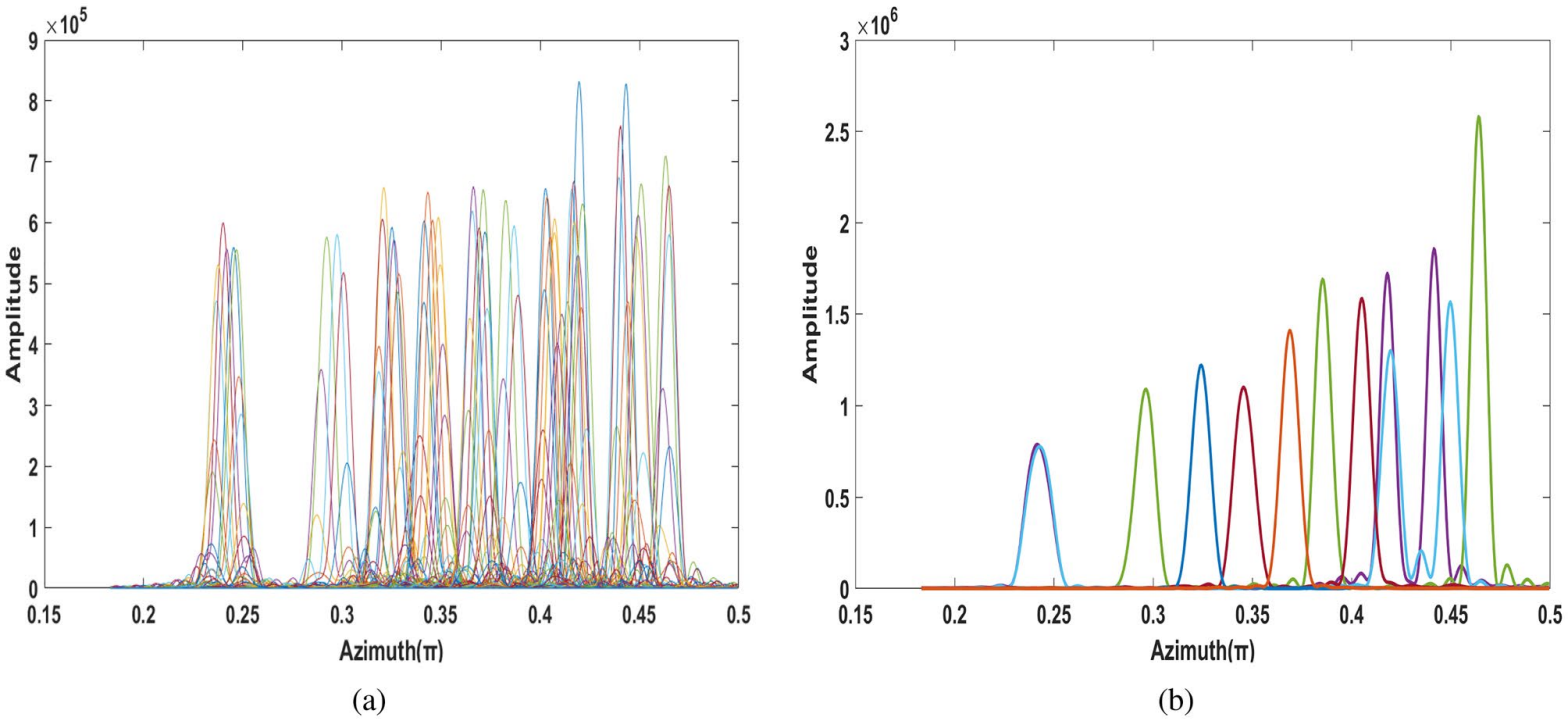
Azimuth profile. (**a**) Azimuth profile before compensation. (**b**) Compensated azimuth profile.

To validate the effectiveness of the proposed imaging method, the aircraft model constituted by multiple ideal scattering pints is utilized for simulation experiments, as shown in Fig. 9. The simulation parameters are the same as Table 1.

The algorithm proposed in this paper is used to estimate the frequency modulation ratio of the echo signal. According to the estimation results, 500 echoes are are randomly selected to calculate the velocity of the target, and then the broadening and shift of the one-dimensional range profile are compensated. The range profile results of the aircraft are shown in Fig. 10, and only 9 scattering points can be obtained.

On this basis, the minimum entropy is utilized as the measurement standard to adaptively compensate the multi-component LFM signal, as shown in Fig. 11. It can be seen that there are 11 scattering points on the compensated azimuth profile.

**Figure 12:**
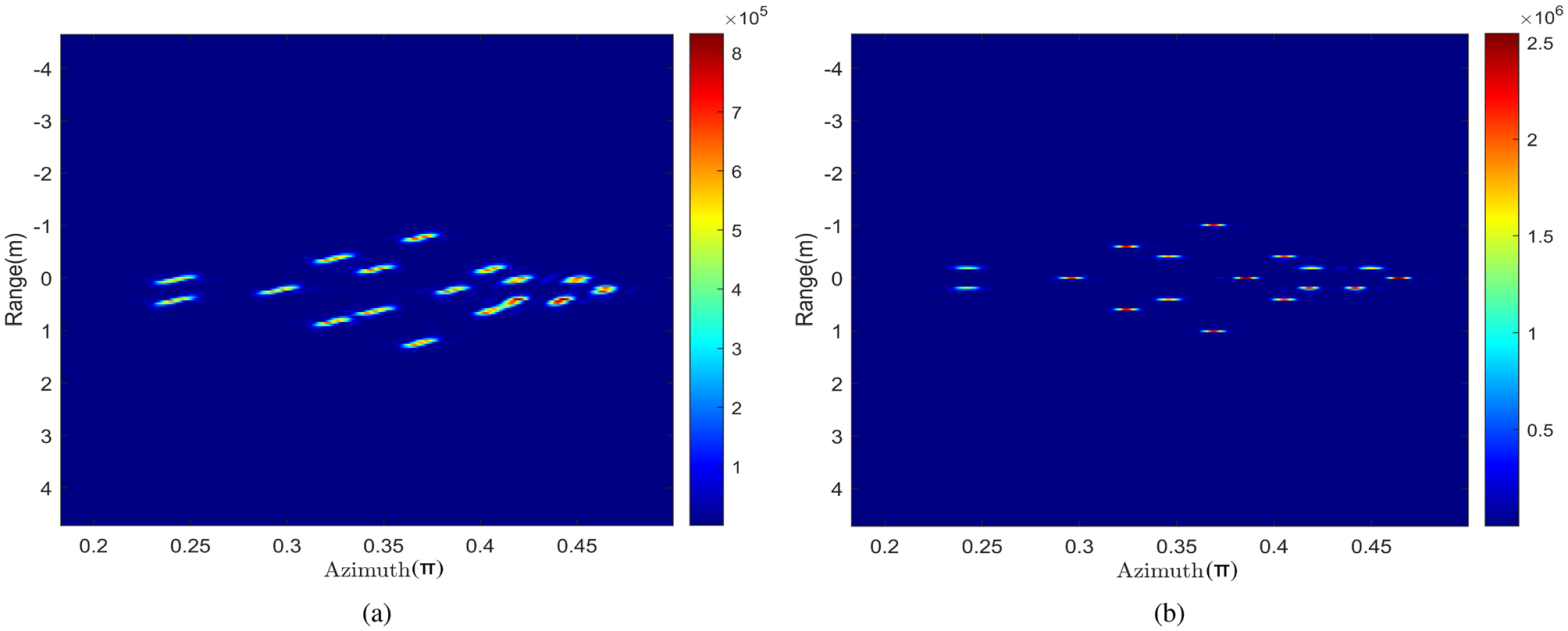
Comparison of two-dimensional imaging of the target. (**a**) Uncompensated 2‐D image. (**b**) The compensated 2‐D image.

**Figure 13:**
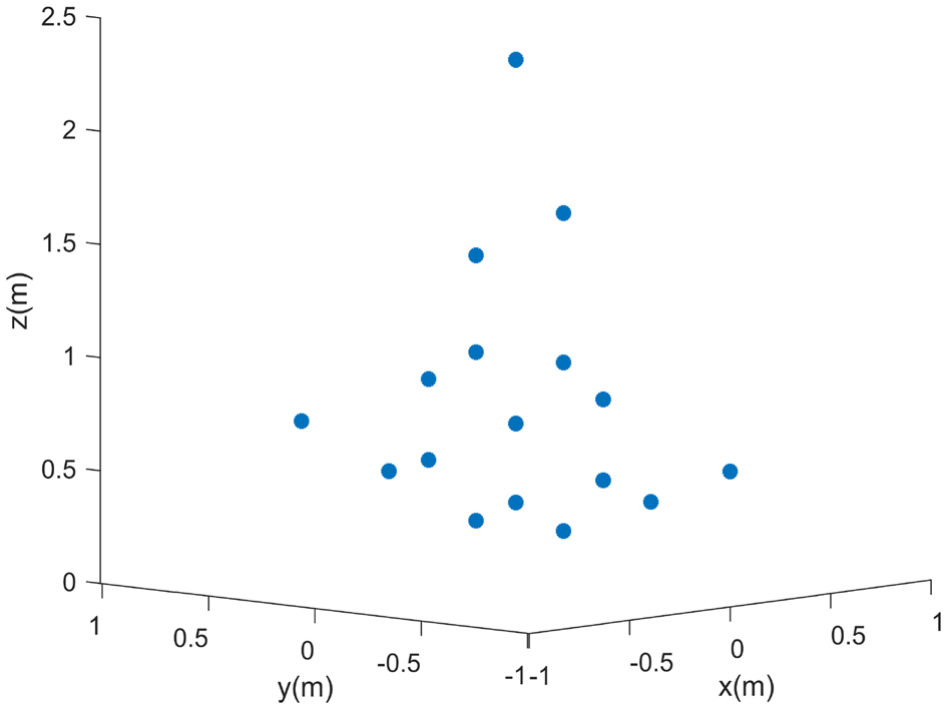
The 3D reconstructed imaging result of the point targets.

The two-dimensional information distribution of scattering points is obtained by combining the range and azimuth image information. The image focusing effect is significantly improved after algorithm compensation, as shown in Figs. 12 and 13 is the final target 3D information reconstruction diagram. The average position error of 17 scattering points is 0.19%,which can be seen that the target’s positions can be reconstructed with high precision.

## Conclusion

A VEMW-based high-speed maneuvering targets ISAR imaging method design for eliminating range profile broadening and azimuth profile blur is proposed in this paper. The movement speed of the target is obtained by estimating the frequency modulation rate of the echo signal. Then adaptive compensation based on minimum entropy effectively eliminates the ambiguity of azimuth profile. Finally, theoretical analysis and simulation results verity the effectiveness of the proposed algorithm. The further work will focus on the VEMW-based parameter acquisition technology for high-speed targets.

## Data Availability

The datasets used and/or analysed during the current study available from the corresponding author on reasonable request.
